# Novel breath biomarkers identification for early detection of hepatocellular carcinoma and cirrhosis using ML tools and GCMS

**DOI:** 10.1371/journal.pone.0287465

**Published:** 2023-11-15

**Authors:** Noor ul Ain Nazir, Muhammad Haroon Shaukat, Ray Luo, Shah Rukh Abbas

**Affiliations:** 1 Atta-Ur-Rahman School of Applied Biosciences, National University of Sciences and Technology (NUST), Islamabad, Pakistan; 2 Department of Electrical Engineering and Computer Science, The Henry Samueli School of Engineering, University of California, Irvine, Irvine, CA, United States of America; 3 National Agriculture and Research Center (NARC), Islamabad, Pakistan; 4 Departments of Chemical and Biomolecular Engineering, Materials Science and Engineering and Biomedical Engineering, the University of California, Irvine, Irvine, CA, United States of America; 5 Department of Molecular Biology and Biochemistry, School of Biological Sciences, University of California, Irvine, Irvine, CA, United States of America; Cranfield University, UNITED KINGDOM

## Abstract

According to WHO 2019, Hepatocellular carcinoma (HCC) is the fourth highest cause of cancer death worldwide. More precise diagnostic models are needed to enhance early HCC and cirrhosis quick diagnosis, treatment, and survival. Breath biomarkers known as volatile organic compounds (VOCs) in exhaled air can be used to make rapid, precise, and painless diagnoses. Gas chromatography and mass spectrometry (GCMS) are utilized to diagnose HCC and cirrhosis VOCs. In this investigation, metabolically generated VOCs in breath samples (n = 35) of HCC, (n = 35) cirrhotic, and (n = 30) controls were detected via GCMS and SPME. Moreover, this study also aims to identify diagnostic VOCs for distinction among HCC and cirrhosis liver conditions, which are most closely related, and cause misleading during diagnosis. However, using gas chromatography-mass spectrometry (GC-MS) to quantify volatile organic compounds (VOCs) is time-consuming and error-prone since it requires an expert. To verify GC-MS data analysis, we present an in-house R-based array of machine learning models that applies deep learning pattern recognition to automatically discover VOCs from raw data, without human intervention. All-machine learning diagnostic model offers 80% sensitivity, 90% specificity, and 95% accuracy, with an AUC of 0.9586. Our results demonstrated the validity and utility of GCMS-SMPE in combination with innovative ML models for early detection of HCC and cirrhosis-specific VOCs considered as potential diagnostic breath biomarkers and showed differentiation among HCC and cirrhosis. With these useful insights, we can build handheld e-nose sensors to detect HCC and cirrhosis through breath analysis and this unique approach can help in diagnosis by reducing integration time and costs without compromising accuracy or consistency.

## Introduction

Hepatocellular carcinoma (HCC) is the most common type of liver cancer and the fourth leading cause of death globally. HCC is still a health problem around the world, and a million new cases are expected by 2025 [1]. Moreover, it is the most common type of liver cancer and accounts for 90% of cases of liver diseases, while cirrhosis is the root cause of HCC [2]. Chronic viral hepatitis B or C, alcohol, and toxins like aflatoxin and pyrrolizidine alkaloids are all linked to HCC and cirrhosis. These risk factors cause oxidative stress (ROS) and cause changes in the liver’s molecular mechanisms [3]. The pathophysiology of HCC is the failure of the cytochrome poly substrate-450 (CYP450) enzyme, which initiates molecular processes that increase the risk of HCC [4]. Ultrasound scans (US), alpha-fetoprotein (AFP) levels, and liver biopsies can be used to diagnose HCC lesions [5]. Data from AFP and liver biopsies showed 30% and 70% of liver cancer patients had depraved outcomes [6]. Early cancer detection is a very active area of research, and for good reason: it improves patient outcomes and lowers the cost of treatment. However, US, MRI, and AFP screening are unable to diagnose the disease at earlier stages. Moreover, these techniques also failed to differentiate the closely related diseases like liver cirrhosis and HCC [7].

Finding new biomarkers that can detect HCC and other diseases early is important for lowering the mortality rate [8]. HCC is a vascularized tumor, and CYP450 produces volatile organic compounds (VOCs) as a byproduct [9]. VOCs can be as big as DNA or as small as H2 molecules, and most of the time they can be found in breath [10]. VOCs are a new class of biomarkers that can be found in a wide variety of compounds, and they provide information about the individual’s body condition; either it is in a healthy state or in a diseased condition. A non-invasive breath analysis could find new biomarkers (VOCs) linked to the inflammatory pathways in cancer [11].

Disease monitoring using exhaled breath analysis (EBA) has been the subject of much research. VOCs come from both inside the body; endogenous VOCs and outside through different eatables known as exogenous VOCs, and are found commonly in exhaled breath, sweat, urine, and sometimes in blood. Endogenous VOCs are generated by oxidative stress, inflammation, and microbes in the body. The VOCs are then released into the bloodstream, where they diffuse into the lungs and are expelled out [12]. Oxidative stress and inflammation change the VOC content exhaled in the breath of the diseased organs. An analytical approach known as Gas chromatography and mass spectrometry (GCMS-SPME) is widely used to analyze these VOCs. This GCMS approach is a gold standard for VOC measurement in breath samples because of its low limits of detection, orthogonal data structure, and molecular structural features [13]. However, there are a few drawbacks of GCMS analysis, including this approach required high expertise, time consumption, and chances of error are also present.

**Fig 1:**
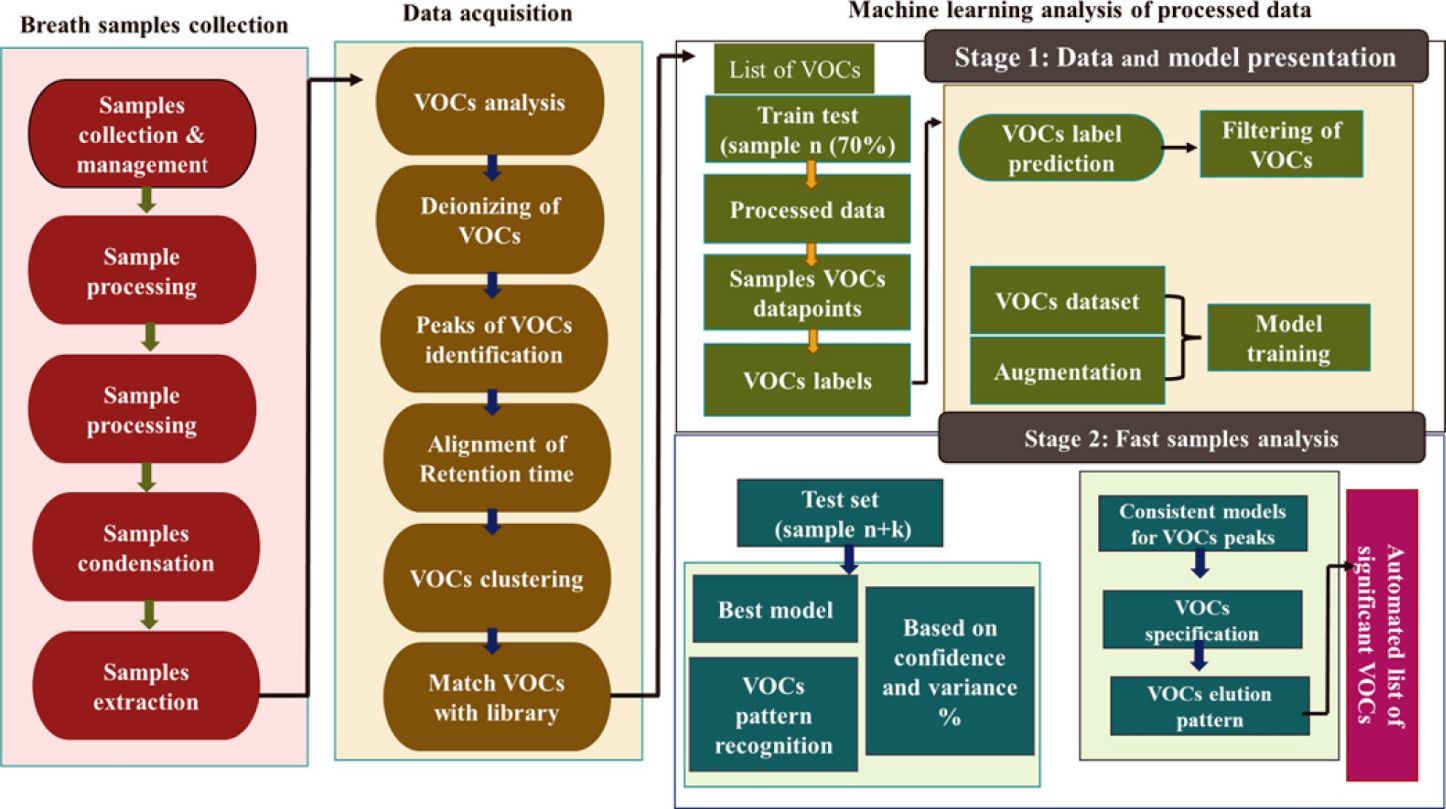
Schematic representation of the study.

**Fig 2:**
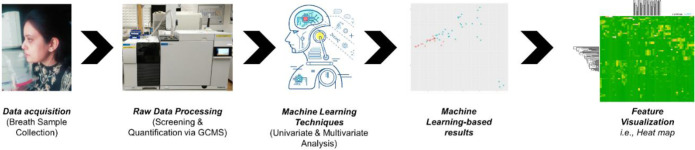
Graphical abstract.

Nowadays, for the early diagnosis of disease accurately through breath biomarkers, machine learning-based algorithms are also used in combination with metabolomics [14]. Multivariate analysis, such as principal component analysis (PCA), partial least discriminant analysis (PLS-DA), and forest mapping are the gold standards in machine learning for VOCs profiling and screening. Moreover, Artificial intelligence (AI) and machine learning (ML) applications have shown significant promise in recent years for improving healthcare and critical care [15]. The present study used a microextraction technique (GCMS-SPME) to collect VOCs from HCC and cirrhosis patients’ breath. For validation of GCMS analysis and accurate classification of these VOCs, in-house R-based machine learning algorithms are used. Another goal of this research is to properly categorize VOCs of closely related medical conditions such as cirrhosis and HCC so that incorrect diagnoses can be ruled out at an early stage. Figs 1 and 2. signifythe schematic and graphical presentation of this study.

## Materials and methods

### Study plan

GCMS-SPME analyzed breath samples [31]. This study compared cirrhotic/HCC patients and healthy people and identified promising VOCs for use in e-nose sensors.

### Patients & subjects

This study comprises cirrhotic, HCC, and healthy subjects. Table 1 showed the demographics of the patient groups. A total of 100 breath samples were taken from patients and healthy volunteers, including students, security guards, hospital staff, and workers.

**Table 1:** Demographic characteristics of study subjects.

Category	Healthy controls	HCC patients	Cirrhotic patients
Total number (n)	30	35	35
**Characteristics**			
Age (mean ±SD)	43 ± 7.6	57.13+43.4	57.1+46.2
Gender (F/M)	12/18	12/23	12/23
Smokers/Non-smoker	10/20	14/21	18/17

#### Inclusion criteria

Untreated hepatitis B & C patients were included, and their diseases were confirmed cytologically and histologically. Cirrhosis and HCC patients, drinkers, smokers/nonsmokers both, and individuals on fasting were included in this study.

#### Exclusion criteria

Pregnant women, people under 18, and those with hepatitis A, renal infections, lung disorders, stomach difficulties, heart ailments, and various cancers were excluded.

### Ethics statement

Protocol # 121 was approved by the National University of Sciences & Technology (NUST) Institutional Review Board (IRB) committee. Before the sample collection, patients or their attendants signed a consent form. The supplementary data (S1 and S2 Text in S1 File) represents the IRB and questionnaire format, which was signed for this study.

### Breath sampling procedures

Patients were given three instructions. 1) Inhale deeply; 2) hold for 5 seconds; 3) exhale slowly into 0.5-liter tedlar bags and 20 ml glass tubes for each patient. All vials were sterilized, and nitrogen flushed.

### GCMS for VOCs extraction and analysis

The VOCs were analyzed by using GCMS with few modifications in the protocol [22]. A pre-conditioned SPME fiber with GCMS was used to analyze breath before measuring VOCs. Before being exposed to the gas phase, the SPME fiber (PDMS) was first placed in the headspace (HS) mode to allow the analytes to be absorbed. The target VOCs responded most to the 75 pm CAR/PDMS fiber. The SPME fiber was placed in the tedlar bags for 15–20 minutes to properly absorb the VOCs. Following the extraction time, it was heated to 250°C for 5 minutes in the chromatograph injector to aid in the desorption of the extracted VOCs. After 15 minutes of extraction, the fiber was immediately exposed for 5 minutes at 250°C in the GC injector. Thermal desorption of the analytes required three minutes. A Shimadzu GCMS-QP 2010 was utilized with a 30 m, 0.25 mm internal diameter, 0.5 m thick, 5% phenyl methyl siloxane capillary column to measure VOCs. High-quality helium gas C60 (China) flowed at 1.0 ml/min. The oven was held at 40°C for 2 minutes, then increased 5°C/min to 200°C (held for 5 minutes), then 280°C (held for 1 min). The transfer line was 280°C, the manifold temperature was 40°C, and the trap was 180°C. Six scans per minute covered 40–400 m/z in this study. 50A emission current and relative electron multiplier mode were used to autotune the method. The maximum ionization time was 25,000 seconds at the rate of 35 m/z.

### Data analysis of VOCs

In this study, VOCs were screened out by GCMS analysis, and a comparison between HCC, cirrhotic patients, and control VOCs was performed by using univariate and multivariate analysis. Significant VOCs were analyzed by using unsupervised and supervised Machine learning (ML) models including principal component analysis (PCA), Random Forest mapping (RF), and Gini mean score. These methods were used to reduce the original huge data set’s variables. To assess model sensitivity, specificity, and diagnostic accuracy the leave-one-out cross-validation model (LOOCV) model was put up in R software. We used mean classification accuracy, sensitivity, specificity, and balanced accuracy (BA) to evaluate the model’s prediction performance. In this study, all analyses were run on IBM SPSS 20.0, Minitab, and R software.

## Results

### Patients demographics

This study includes a total of 70 diseased patients and 30 healthy individuals. All individuals consist of three groups such as cirrhotic (n = 35), HCC (n = 35) patients, and healthy (n = 30) individuals. The average ages of healthy, HCC, and cirrhosis patients are 43.0±7.6, 57.1±43.4, and 57.1±46.2. Males account for 65% of all participants, and females are responsible for 35%. This study also examined smoking, alcohol use, and Hepatitis-B/C virus infection’s effect on the disease worsening. The statistical results of patient demographics are shown in Tables 1 & 2.

**Table 2:** Statistical analysis of HCC & cirrhotic groups.

Cirrhotic	St-Deviation	Chi-square	t-Value	Mean	p-values	St-Deviation Mean
Age	11.28 ± 10.09	0.002	5.106 ± 5.219	57.1 ± 46.2	0.33	1.44 ± 1.57
Gender	0.46 ± 0.49	0.19	-1.064 ± 1.05	1.31 ± 1.41	0.59	0.059 ± 0.077
Smoking	0.500 ± 0.460	0.093	-1.53 ± 1.55	1.55 ± 1.70	0.003	0.064 ± 0.071
Drinking	0.12 ± 0.0000	0.598	-0.818 ± 1.000	1.98 ± 2.0	0.099	0.016 ± 0.000
HBV	0.387 ± 0.156	0.04	-2.44 ± 2.820	1.89 ± 1.97	0.000	0.049 ± 0.024
HCV	0.484 ± 0.40	0.000	-4.850 ± 5.040	1.3 ± 1.8	0.000	0.61±0.062
**HCC**	**St-Deviation**	**Chi-square**	**t-Value**	**Mean**	**p-values**	**St-Deviation Mean**
Age	10.2 ± 7.6	0.021	6.11 ± 6.27	57.13 ± 43.4	0.088	1.70 ± 1.39
Gender	0.47 ± 0.49	0.380	-0.553 ± 0.551	1.33 ± 1.40	0.293	0.07 ± 0.09
Smoking	0.50 ± 0.40	0.011	-2.61 ± 2.66	1.50 ± 1.80	0.000	0.084 ± 0.074
Drinking	0.16 ± 0.00	0.54	-0.912 ± 1.000	1.97 ± 2.00	0.065	0.027 ± 0.000
HBV	0.37 ± 0.00	0.021	-2.41 ± 2.64	1.833 ± 2.00	0.000	0.062 ± 0.00
HCV	0.47 ± 0.00	0.000	-7.6 ± 8.36	1.33 ± 2.00	0.000	0.079 ± 0.00

Pearson chi-squared and probability tests were used to confirm the differences in VOCs among all groups. Table 1 shows the demographics of both groups, while Table 2, analyzes the confounding factors such as gender, age, smoking, alcohol use, and hepatitis B&C. All covariates (smoking & hepatitis B&C) had significant *p* values (<0.05) when compared to the control group, except for gender, alcohol consumption, and age. Smoking and hepatitis (B&C) have *p* values less than <0.05, indicating they substantially impact disease condition and severity.

### VOCs screening

#### Screening with SPME-GCMS

**Fig 3:**
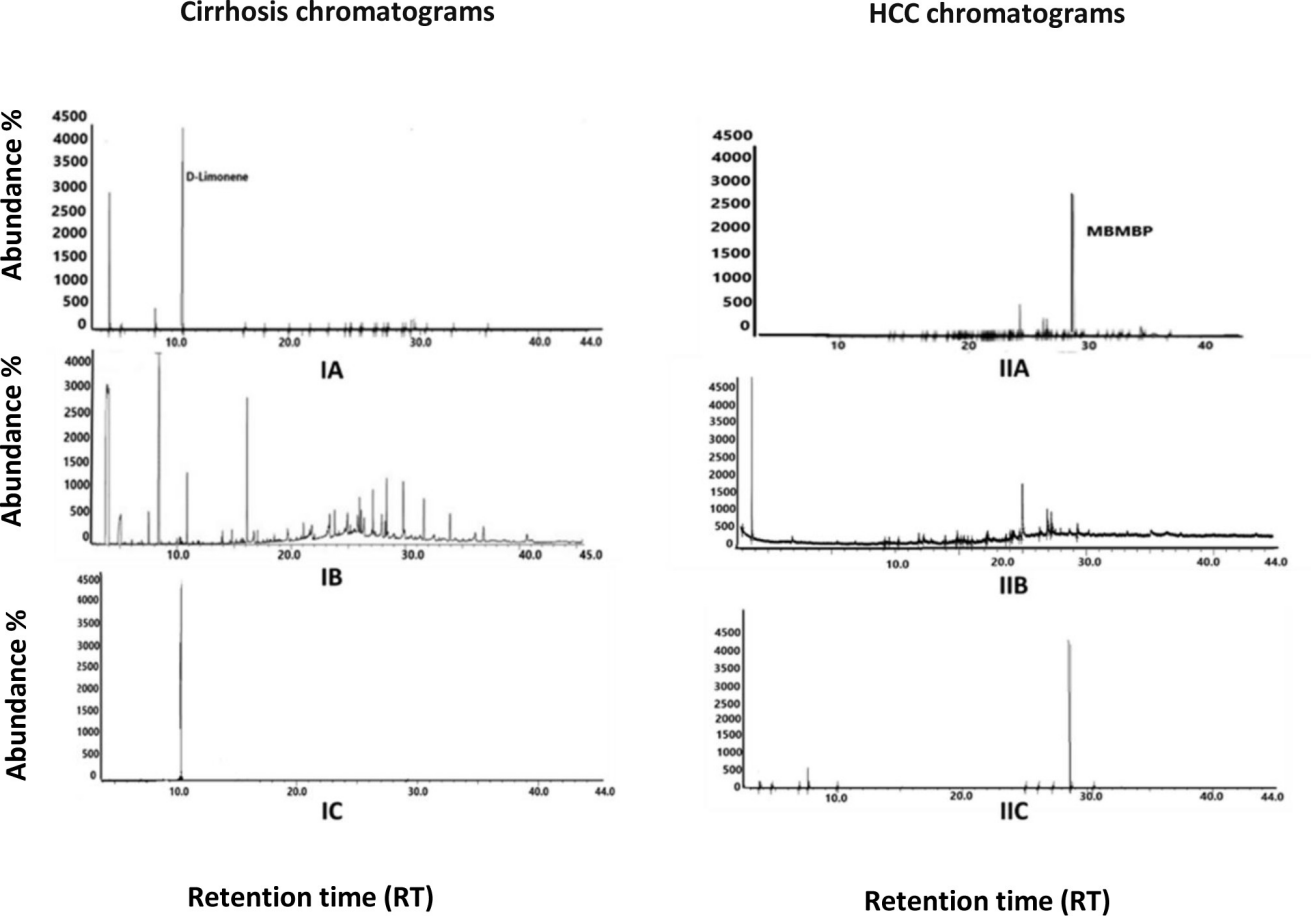
Comparison of GCMS chromatographs of Cirrhosis patients, controls, and standard solution (IA, IB&IC) & HCC patients, control and standard solution (IIA, IIB & IIC).

In this study, GCMS spectral library detected endogenous or exogenous VOCs in exhaled breath samples from cirrhotic, HCC, and healthy volunteers. Numerous VOCs were screened out in the GCMS chromatograms of HCC and cirrhotic patients, but very few were essential based on area percentage and prevalence rate. The significant biomarkers in the GCMS analysis are the following; Phenol, 2,2’-methylenebis[6-(1,1-dimethyl ethyl)-4-methyl (MBMBP) (compound 1), sulfurous acid hexyl octyl ether (compound 2), 1,3-benzene dicarboxylic acid (compound 3) and toluene (compound 4) in HCC breath samples whereas 3-hydroxy-2-butanone (compound 5), methane sulfonyl chloride (compound 6), styrene (compound 7), di-n-octyl phthalate (compound 8), and d-limonene (compound 9) in the cirrhotic breath samples. The peak location of these significant breath biomarkers according to retention time (RT) interval, is shown in (S1 Table in S1 File). In Fig 3. Comparison of GCMS chromatographs of Cirrhosis patients, controls, and standard solution (IA, IB & IC) & HCC patients, control and standard solution (IIA, IIB & IIC) areperformed. The D-Limonene peak was visualized at a retention time of 10.74 minutes in all graphs, which indicates the authenticity of the results. However, this peak was absent in the control chromatogram, whereas for the verification of this peak, the standard solution of D-limonene was run at the same GCMS parameters. Similarly, the graphs (IIA & IIC) showed phenol, 2,2’-methylene bis [6-(1,1-dimethyl ethyl)-4-methyl (MBMBP) peak at 27.4 minutes in the HCC group and standard solution, whereas this peak is absent in the GCMS graph of control individual (IIB). From the graph of a standard solution of MBMBP, it can be justified again that the MBMBP peak is significant and confirmed at 27.4 minutes. From this study’s results, it can be suggested that MBMBP is for HCC, and d-limonene breath biomarkers for cirrhosis could be proven as diagnostic biomarkers. Other compounds mentioned in this studywere also found as obvious biomarkers in the GCMS results. However, the individual graph of each biomarker is difficult to show here in Fig 3. To corroborate the validity of the data, more reliable results were obtained using machine learning analysis, such as principal component analysis.

### Unsupervised machine learning analysis—Principal multivariate analysis

In this research, multivariate statistical analysis, and machine learning methods (ML), both supervised and unsupervised, were used to study HCC and cirrhotic VOCs. After identifying the significant VOCs through statistical analysis, ML models were used to screen out the potent VOCs stepwise and get the most sensitive VOCs for early diagnosis of HCC and cirrhosis.

### Unsupervised machine learning models

#### Principal ComponentAnalysis (PCA)

**Fig 4:**
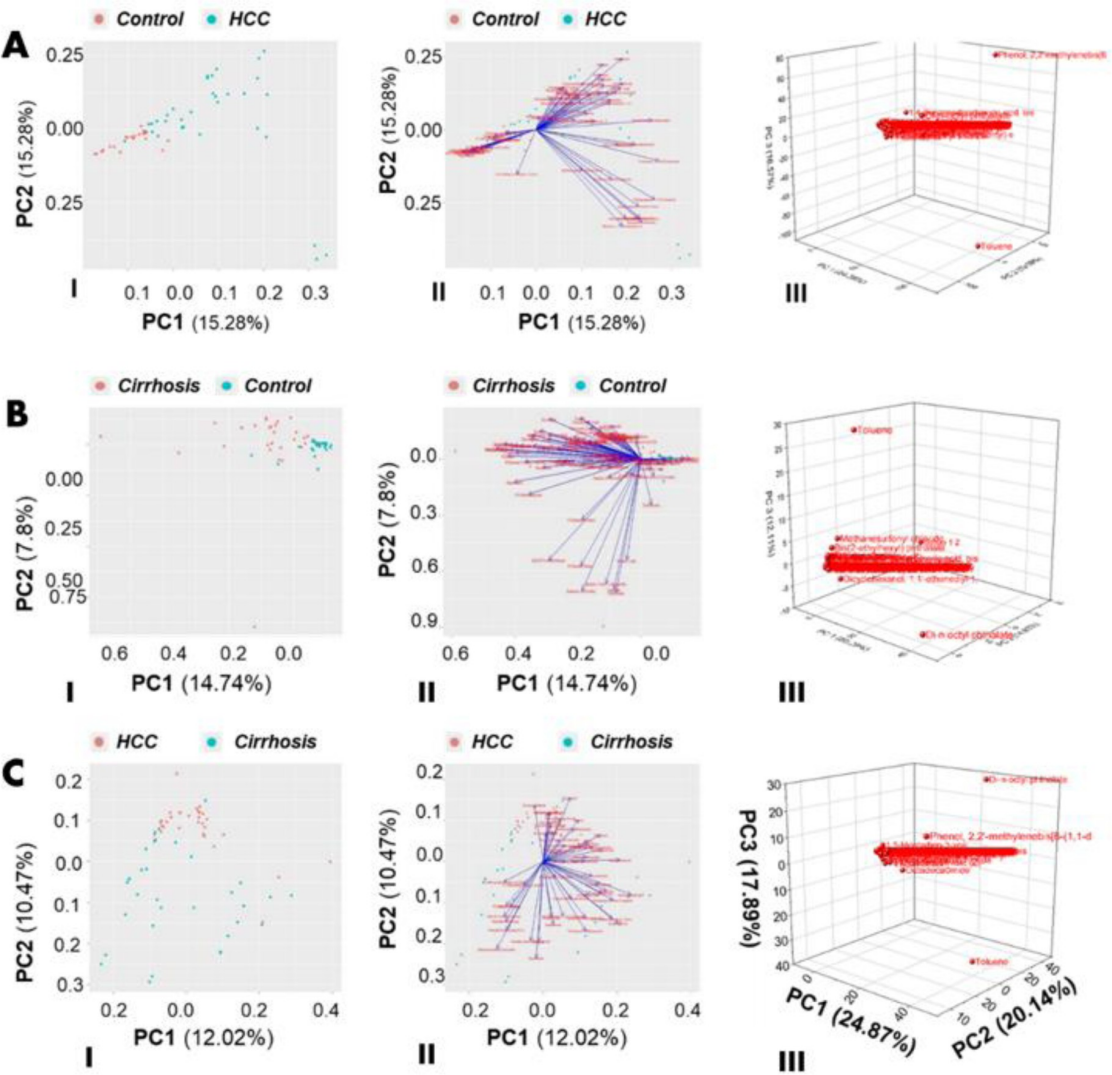
PCA analysis: (A) HCC vs Control (I) Score plot 2D (II) Loading plot (III) Score plot 3D; (B) Cirrhosis vs Control (I) Score plot 2D (II) Loading plot (III) Score plot 3D; (C) HCC vs Cirrhosis (I) Score plot 2D (II) Loading plot (III) Score plot 3D.

This study screened thousands of VOCs using the GCMS approach and further analyzed them by PCA whereasvariance and dimensionality were used to categorize and reduce VOCs in PCA analysis. Fig 4 demonstrated PCA analysis: (A) HCC vs Control (I) Score plot 2D (II) Loading plot (III) Score plot 3D; (B) Cirrhosis vs Control (I) Score plot 2D (II) Loading plot (III) Score plot 3D; (C) HCC vs Cirrhosis (I) Score plot 2D (II) Loading plot (III) Score plot 3D.

#### HCC vs. control

PCA analysis found 54 VOCs in HCC vs. control group. Fig 4(A) shows that PC1 seems to have 18 VOCs and PC2 has 36 in all graphs. Compounds 1 (p<0.001), 2 (p<0.05), 3 (p<0.05), and 4 (p<0.005) with positive eigenvalues are the most significant VOCs for HCC. Eigenvalues show correlations between two factors, like biomarkers and disease. Fig 4A(I & III) show 2-D and 3-D PCA score plots for HCC and control VOCs. The 2D plot showed the simple clustering of all screened-out VOCs, while the 3-D plot shows the exact dimension and axis of each biomarker based on PC components. Fig 4(II) showed the PCA loading with PC1 = 15.28% and PC2 = 15.28%. It means PC1 and PC2 VOCs fluctuate substantially. The HCC vs control 2D score plot showed a 15.28% variance in PC1 and PC2. The 3D PCA plot shows PC1 = 24.87%, PC2 = 20.14%, and PC3 = 17.89%. PC1 does have more variance than PC2 and PC3. S1 Fig in S1 File illustrated the scree plots (A) HCC vs control (B) Cirrhosis & control (C) HCC vs cirrhosis.

#### Cirrhosis vs. control

PCA identified 32 VOCs in cirrhosis and the control group, 22 in PC1 and 10 in PC2. Fourteen VOCs were selected out of 32 compounds, based on a variance in PCA analysis. Fig 4B(I &III) shows 2-D and 3-D graphs, with 14.74% variance in PC1 and 7.8% in PC2. So, PC1 in the 2D graph has more variance than PC2, and VOCs in this plot are clustered and separated precisely. The 3-D graph of PCA analysis showed the variance percentages as PC1 = 24.87%, PC2 = 20.14%, and PC3 = 17.89%. PC1 contains compounds 5 (p>0.005), 6 (p>0.001), 7 (p>0.05), 8 (p>0.05), and 9 (p>0.001), while PC3 has compound 4 (p<0.05) in 2D and 3D plots. The score plot of cirrhosis vs control screened out 22 VOCs. Moreover, Fig 4B(II) showed the loading plot of cirrhosis VOCs which indicates considerable positive eigenvalues showed high variances for all these compounds. The cirrhotic VOCs significance is indicated by the scree plot which is illustrated in (S1 Fig in S1 File).

#### HCC vs. cirrhosis combined biomarkers

A total of 52 VOCs were grouped together as PC1 in the PCA 2-D and 3-D analyses for the third group of patients with HCC versus cirrhosis Fig 4C(I & III). In 2D and 3D PCA analyses, PC2 identified 1 HCC and 6 cirrhosis VOCs. The eigenvalues of compounds 1, 4, and 2 were high in HCC patients, while compounds 5, 6, 7, and 9 were significant in cirrhotic patients. In 2-D and 3-D PCA plots, HCC and cirrhosis VOCs are clearly separated. Fig 4C(II) demonstrates HCC loading plots whereas, the 2D score plot of HCC vs cirrhosis reveals 12.02% in PC1 and 10.47% in PC2. Fig 4C(II) loading plot has the same variance as the 2-D score plot. In Fig 4C(III), the 3-D plot showed the score plot with these variance percentages: PC1 = 24.87%, PC2 = 20.14%, and PC3 = 17.89%. This reflects earlier group results indicating PC1 compounds have high variance and significance relative to PC2 and PC3. The variation captured by the PCA analysis in HCC vs. cirrhosis group is also illustrated by the scree plot, as shown in (S1 Fig in S1 File).

#### Heat maps

**Fig 5:**
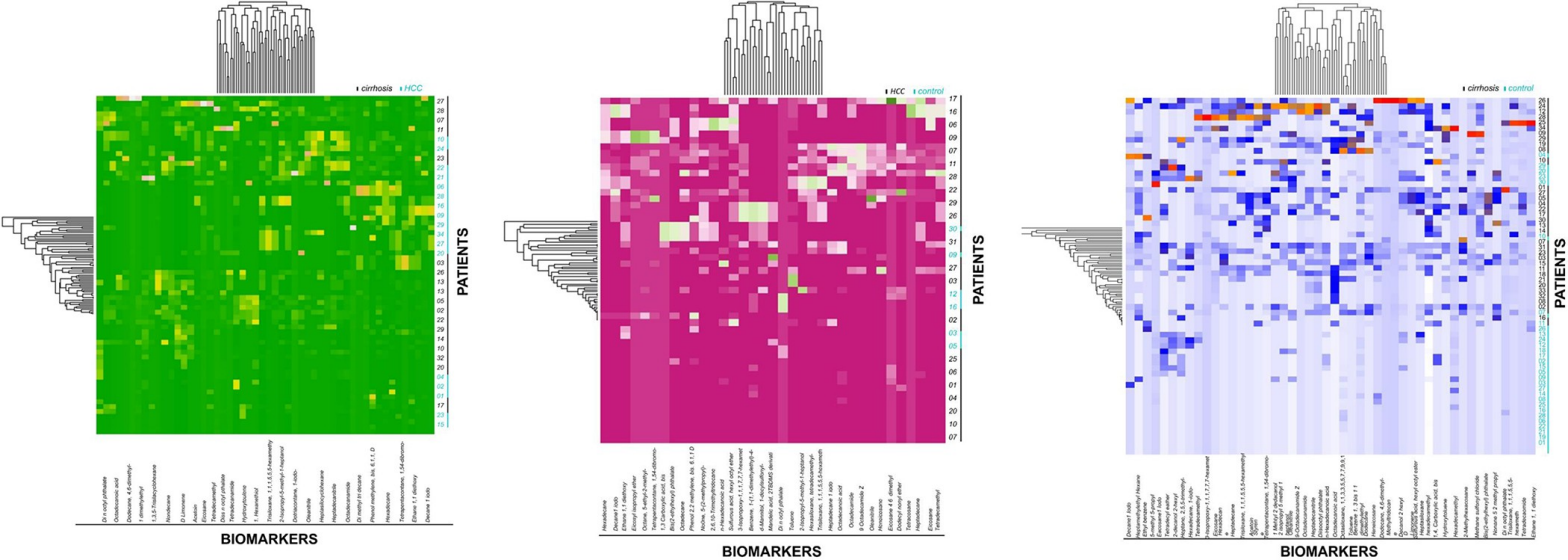
Comparison of hybrid heat maps among 3 groups (A) Cirrhosis vs control (B) HCC vs control (C) HCC vs cirrhosis.

Fig 5 illustrated the comparison of hybrid heat maps among 3 groups (A) Cirrhosis vs control (B) HCC vs control (C) HCC vs cirrhosis. Fig 5(A) hybrid heat map showed metabolites distinguished well among cirrhosis and controls. Compounds 4, 5, 6, 7, 9, 10, and 11 have strong discriminating power with an accuracy range of 79% to 93%. Fig 5(B) showed the hybrid heatmap of HCC vs. control groups, indicating the highest separability of VOCs such as compounds 1, 2, 4, 8, 10, and 11. Fig 5(C), showed the metabolites of HCC vs. cirrhosis and cross-verified the prior results by highlighting the same VOCs. This study demonstrated great group stability with negligible drift and fluctuation.

### Supervised machine learning models

#### Gini mean score plot: HCC vs. control

**Fig 6:**
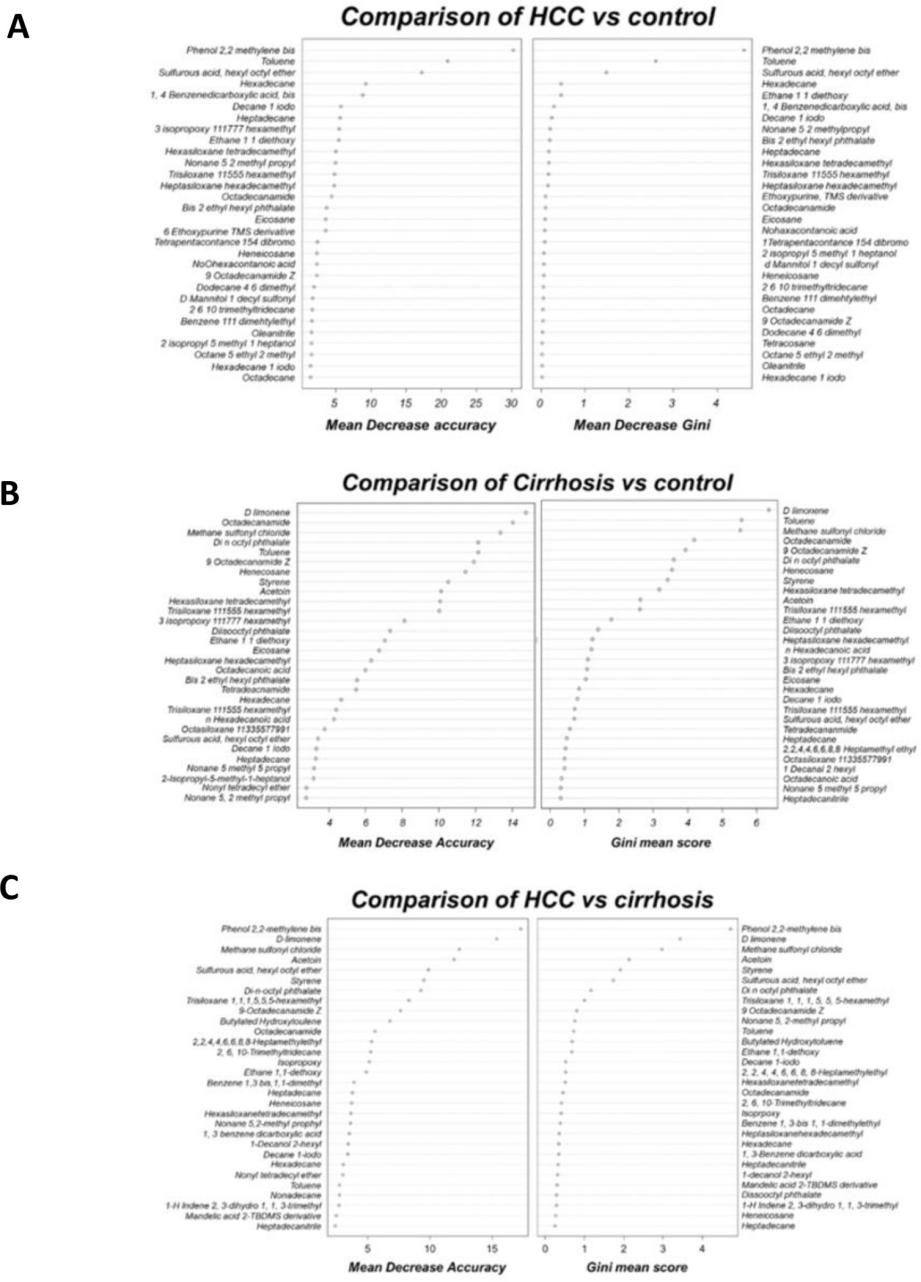
Forest model, comparison of Gini means the decreased frequency of (A) HCCs vs control (B) Cirrhosis vs control (C) HCC vs cirrhosis.

Gini Mean Score Plot ensures more accurate results overall, with minimum errors. This group showed NPV 85% precise and PPV values were 100%. Based on these NPV values in our model, negative metabolites were successfully recognized. The kappa classification accuracy in this group was 86.4%, the trees used were 800n, and the confidence level of this group was 95%. The model’s *p*-value was <0.005, demonstrating its significance. This model’s 1.89% error rate is quite low compared to other ML analyses, indicating it’s a perfect model. Fig 6 demonstrated the Forest model, a comparison of Gini means the decreased frequency of (A) HCCs vs control (B) cirrhosis vs control, and, (C) HCC vs cirrhosis. Fig 6A shows that compounds 1, 2, and 4 were the most discriminating biomarkers in this group. Dodecyl nonyl ether (compound 11), hexadecane-1-Modo (compound 12), octane ethyl 2 methylene (compound 13), eicosyl isopropyl ether (compound 14), and benzene 1,1,1 dimethyl ether (compound 15) are less important biomarkers in this model.

#### Cirrhosis vs. control

The model’s best test group was cirrhosis vs. control, with 100% accuracy and precision. This model’s PPV and NPV were 85% and 100%, with 93.3% accuracy. The NPV was 100%, showing that most cirrhosis VOCs were also identified in other disease groups. VOCs specific to cirrhosis had an 85% effect on the disease group. This model has 100% accuracy for BA, NPV, and kappa classifications with a *p*-value <0.00005, indicating high significance. This model’s error rate was 1.56%, and the group’s confidence level is 95%, suggesting its stability. Fig 6B compares cirrhosis and control compounds and it is found that compounds 5, 6, 9, 8, and 10-(9-octadecamide Z) showed a good mean decreasein accuracy in this model.

#### HCC vs. cirrhosis

The overall classification accuracy in this group was 89.4%, 100% sensitive, 80% specificity, and 90% accuracy. This model achieved 81.8% PPV and 100% NPV with *p*-values <0.0005. This model used five mtry, 800 n-trees, and showed a 4.4% error rate. Fig 6C showed that HCC biomarker compound 1 and cirrhotic biomarkers 3, 5, 6, and 9 were significant in comparing both disease groups.

#### Test score

LOOCV (Leave One out Cross-Validation) is a cross-validation approach in which each observation represents the validation set. Random forest model sensitivity and specificity for HCC vs. control is 80%. In cirrhosis vs control, the model’s sensitivity was 60–80% and specificity was 100%. The HCC vs. cirrhosis group has 100% specificity, however, sensitivity was 60% to 80%. These results revealed overlap accuracy with the leave-one-out cross-validation (LOOCV) model for the three groups: (HCC against control, cirrhosis versus control, and HCC versus cirrhosis) are 93%, 100%, and 89%, respectively. The performance and validation of the RF Model are explained in the S1 Data.

#### The discriminant power assessment

(S2 Fig in S1 File) illustratesROC curves for the diagnostic accuracy of breath biomarkers (VOCs) HCC and cirrhosis. HCC VOCs: Compound 1, compound 4, and compound 2 have ROC (AUC) curves with a sensitivity of 0.99%, 0.97%, and 0.91%, respectively. Compounds 5, 6, 9, 7, and 10 (9-octadecanamide-Z) are major VOCs in cirrhosis, with a diagnostic sensitivity of 0.97%, 1%, 0.99%, 0.91%, and 0.98%.

## Discussion

### VOCs analysis through GCMS

Melanoma protein and gene alterations promote cancer and induce membrane peroxidation [16]. This disrupts blood chemistry, alters liver function, and emits VOCs in breath [17]. VOCs are known for non-invasive, low-cost early disease detection. Metabolomics combines several VOC extraction headspace techniques with statistical analysis to find novel biomarkers in sweat, breath, blood, and urine to diagnose disease. Comparing cancer patients to healthy people, exhaled breath showed significant VOCs [18]. Catarina L. Silva et al. (2016) used GCMS to study VOCs in breast cancer and found potential biomarkers [19]. Stavropouloset et al. (2021) identified 1-octene with an aldehyde in lung cancer patients’ breath using GCMS [20]. The current study also used GCMS to compare HCC and cirrhosis breath samples to healthy controls. This study also examined whether specific breath VOCs observed in liver cirrhosis and HCC patients might be useful for differential diagnosis at early stages. For breath VOC analysis, subjects were classified into HCC, cirrhotic, and healthy groups. From the results, it is found that various VOCs are more significant in HCC patients than in cirrhotic patients and healthy individuals.

Changes in VOCs amounts are attributed to the liver’s lipid metabolism and cellular amino acids. Compound 1, compound 2, and compound 4 peaks were revealed as major VOCs in the HCC GCMS chromatogram. These VOCs have a higher peak area and height than controls. Compounds 5, 6, 7, 8, and 9 were revealed in high amounts in cirrhotic patients’ breath samples. The above analysis was based on all patients’ mean VOC accuracy. The VOCs were chosen based on the percentages of area and peak heights. The VOC was found to be significant in all HCC patients and was then compared in all cirrhotic patients and healthy individuals’ breath samples. The VOCs were chosen based on mainly two factors: first, the prevalence rate of specified VOC in the required diseased patients, and second, the comparison of that VOC in all other groups’ results. The Venn diagram program was used to refine all the GCMS results in this study.

In the present work, numerous statistical analyses and supervised and unsupervised machine learning (ML) models were also applied to the GCMS data to refine the results and specifically identify the potential biomarkers based on classification and clustering. ML models allow for the distinction between cirrhotic and HCC stages to be observed. Both cirrhosis and HCC are liver diseases, and it is plausible that the former is the underlying cause of the latter. It has been noted that most individuals with cirrhosis die in the advanced stages of the disease, long before the development of HCC. Thus, there exist distinctions between the two diseases, and doing so is vitally crucial. In this investigation, there was just a handful of VOCs that stood out as significantly differentiating between the two disease groups. However, there is a volatile organic compound (VOC) called Methane sulfonyl chloride that is more prevalent in cirrhotic individuals but is seen in few of the HCC patients, therefore additional research is needed into this biomarker. It is clear from this study that the emission of this VOC molecule begins in the early stages of cirrhosis and persists through the last stages of HCC. Multivariate analysis, therefore, is required for the investigation and confirmation of the theory to provide more reliable results for this study.

### Multivariate analysis

#### Unsupervised machine learning models—PCA analysis

In one study, PCA analysis found seven metabolites with 93.59% sensitivity and 71.62% specificity for breast cancer. Seven biomarkers from PCA analysis predict DCIS with 80% sensitivity and 100% specificity [21]. Principal component analysis (PCA) based on clustering and classification exposed HCC and cirrhotic-specific biomarkers. From the PCA analysis, as shown in Fig 4A(I-III), three HCC VOCs, compounds 1, 2, and 4, were found significant, with sensitivity and specificity of 80% and 100% respectively. Five VOCs of cirrhosis, compounds 5, 6, 7, 8, and 9, were found in the PCA analysis with 100% sensitivity and 80% specificity compared to the control group Fig 4B(I- III). PCA results in HCC and cirrhotic patients show high sensitivity and unique clustering of VOCs. PCA clustering classified both groups’ VOCs in Fig 4C(I-III), whereas the 3-D PCA plot shows the exact dimensions and angles of each breath biomarker for each PC component.

In all groups, PC1 had a larger variance than PC2 and PC3. PCA analysis of all 3D graphs shows that VOCs away from the original line of the graph have a substantial impact on disease progression, Compounds 1 and 4 demonstrated a larger impact on HCC progression in 3D PCA analysis, shown in Fig 4A(III) because they are away from the origin line and these compounds are positively correlated with each other. Compounds 4 and 8 in the cirrhosis group had a negative correlation, so if one increased, the other one decreased. Compound 4 is positively correlated with VOCs in the same PC group, while VOCs in the opposite direction are negatively correlated. In the 3D graph of cirrhosis vs. control, displayed in Fig 4B(III), compound 4 is favorably correlated with compounds 6 and 7. However, compound 4 and compound 8 are negatively correlated. Thus, PCA results of the first two patient groups identified crucial biomarkers by reducing data dimensionality, which was cross verified in the third group: HCC vs. cirrhosis, as shown in Fig 4C(III).

#### Hybrid heat maps

In recent studies, significant VOCs of most malignant diseases, such as cancer, Crohn’s disease, ulcerative colitis, and preeclampsia were screened out based on the heat maps analysis [22, 23]. In that study, significant VOCs of most malignant diseases, such as (Crohn’s disease, ulcerative colitis, and preeclampsia), were similar [23]. In the current study, Fig 5A–5C depicts hybrid heat maps showing significant VOC clusters between HCC, controls, and cirrhotic groups. In hybrid heatmaps, patients with similar VOCs were grouped together. In our results, major HCC VOCs in Fig 5(B) are compounds 1, 2, 4, 8, 10, and 11, while significant cirrhosis VOCs in Fig 5(A) are compounds 5, 6, 7, 8, 9, 10, and 11 whereas, compounds 8, 10, and 11 are found significant in both HCC and cirrhosis heat maps, shown in Fig 5(C). Hybrid heat map results will depend on the sensitivity and specificity of the VOCs for their respective disease groups, which are based on variance and interrelation. More analysis is needed to refine the results and identify the most significant VOCs through supervised machine-learning models.

### Supervised machine learning models

#### Random forest mapping & Gini mean score plot

RF mapping is a sensitive supervised machine learning approach. Hershberger et al. (2021) evaluated breath VOCs using a random forest model. In that study, disease VOCs when combined with age and sex helped in the classification of disease conditions more precisely. The RF model in that research predicted acetoin and di-n octyl phthalate with 85% accuracy, 80% sensitivity, and 95% specificity [24]. In this investigation, health conditions were influenced by substantial VOCs found in the patient’s breath. This demonstrates that the VOC levels in breath samples of HCC, cirrhotic patients, and controls vary. Exhaled VOCs’ sensitivity and specificity in RF models were used to predict the state of HCC disease. The VOCs at the top of Fig 6A–6C, had a substantial impact on the disease as they had a high mean decrease accuracy percentage. Overall, RF results are more accurate than other ML model results. The rise in accuracy using RF suggests this method evaluates VOCs and their relevance more accurately.

The Gini mean score plot was also used to screen out significant VOCs based on mean accuracy [25]. RF mapping methods were used to categorize VOCs and find the most important compounds. Only one or two components with high mean decrease accuracy will be considered diagnostic VOCs for each disease. Compounds 1, 2, and 4 of the HCC vs. control group revealed a higher mean decrease in accuracy, indicating that these VOCs are significant for diagnosing HCC disease, as shown in Fig 6A. Compound 1 had a higher mean score than compounds 2 and 4, indicating it was chosen as the diagnostic compound for HCC diagnosis.

Gini means score plot was also used to screen out five significant VOCs from the cirrhotic vs. control group. Compound 9 earned the highest mean decrease accuracy compared to the other VOCs in the cirrhotic group (Fig 6B), hence it is considered the diagnostic compound for cirrhotic patients. Although the benefits of random forests in this context have been noted in prior sections, it is vital to remember that the RF model may find correlations and high sensitivity between VOCs. Similarly, Fig 6C showed the same significant diagnostic VOCs in HCC and cirrhosis groups such as Compound 1 and compound 9, respectively.

#### ROC curves

Zhang et al. (2020) employed ROC curves to identify breast cancer and DCIS. He found that breath biomarkers were 93.59% and 71.62% sensitive and specific whereas the AUC and ROC curves demonstrate a test’s discriminative power. The higher the AUC percentage and the closer the curve to the upper-left corner, the better the tests for disease-healthy discrimination. The AUC curve, which ranges from 0 to 1, indicates a test’s accuracy such as perfect diagnostic test AUC = 1.0, whereas nondiscriminatory test AUC = 0.5 [26, 27].

ROC curves demonstrated in this study, that compound 5 is the primary breath biomarker for liver disease (cirrhosis), with 83.3% sensitivity and 91.7% specificity. The AUC curves of cirrhotic biomarkers provided in S1 Fig in S1 File demonstrate the sensitivity of the compounds 9, 5, 7, 6, and 10 are 100%, 94%, 94.5%, 98.7%, and 95% respectively, exhibiting high diagnostic accuracy. In contrast, HCC biomarkers such as compounds 1, 2, 3, and 4 demonstrated 100%, 93%,95%, and 98% diagnostic accuracy or sensitivity, as shown in (S2 Fig in S1 File). This study’s ROC curves were based on statistical VOC area percentages.

The metabolization of VOCs in the human liver. Saturated hydrocarbons are converted to alcohol in healthy people by the P450 enzyme. This enzyme was reduced in the body due to liver injury and disrupts exhaled VOCs. Oxidation stress (ROS) and lipid peroxidation are two mechanisms for free radical-driven VOC emission in the breath. Chronic hepatic conditions reduce CYP450, liver functional units, portal blood flow, and liver extraction capacity [28, 29]. Although the molecular mechanism is uncertain [30], interleukin 6 (IL-6) could be key.

**Fig 7:**
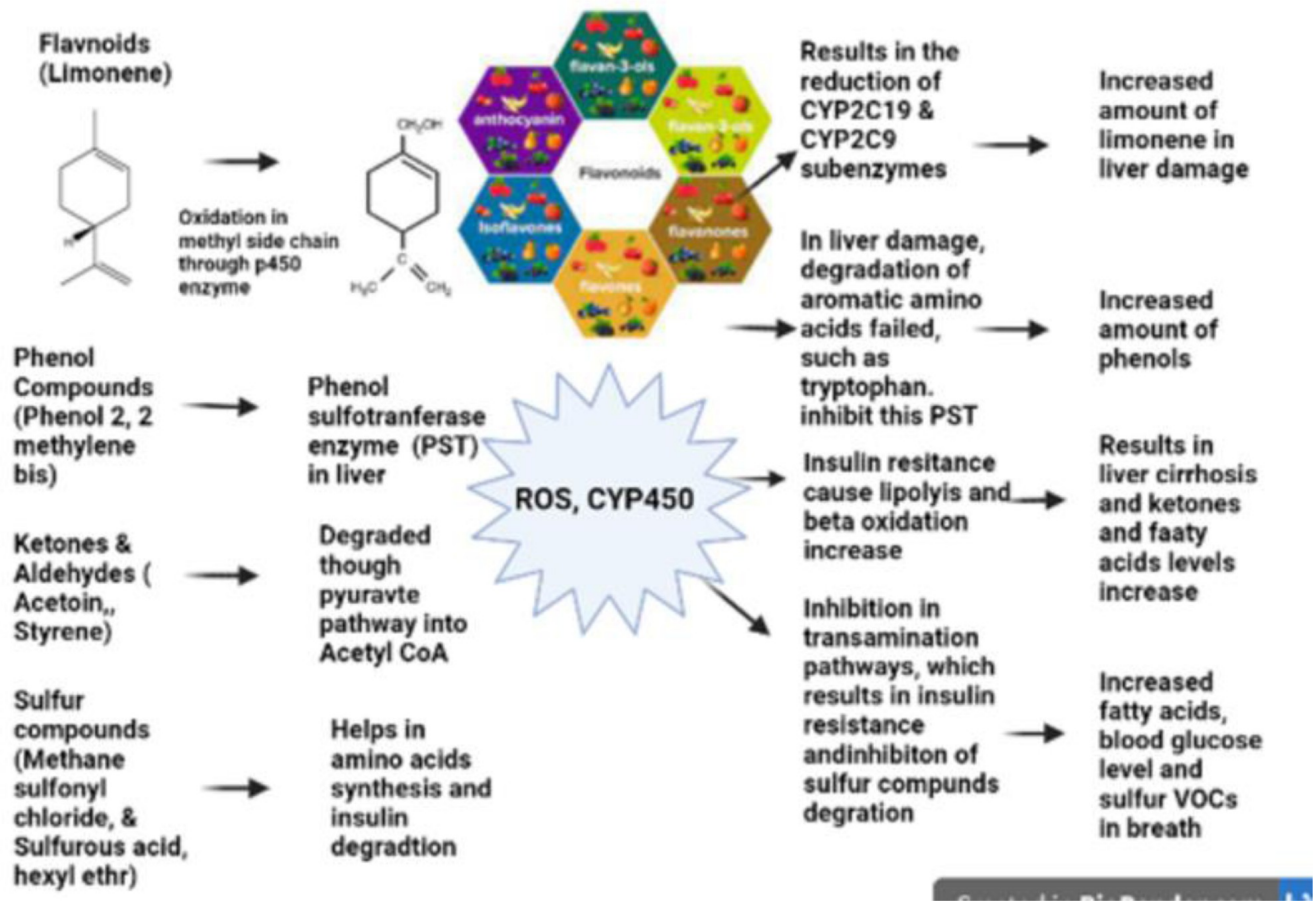
Flow chart of VOCs metabolization in human liver.

Our study supports the concept in the literature that differences between healthy control VOCs and liver disease patients are linked to CYP450, CYP2E1 enzyme, and CYP2B6 isoform activity alteration in the body due to liver injury. Fig 7 depicted theflow chart of VOCs metabolization in the human liver whereas the (S1 Table in S1 File) elaborated the statistical analysis of HCC and cirrhotic VOCs with their concentrations and *p*-values found in this study.

#### Sulfur transamination in liver

Incomplete transamination of sulfur-containing amino acids in the liver through the P450 enzyme produces sulfur-containing molecules. Sulfur compounds are associated with liver damage, giving cirrhotic patients a unique odor [31]. In healthy people, the P450 enzyme converts saturated hydrocarbons to alcohols, however, in Liver damage this enzyme gets reduced and disturbed the number of VOCs in a breath. Oxidative stress and lipid peroxidation are two free radical-driven mechanisms overall for VOCs emission, as shown in Fig 7. In this study, the sulfur-containing breath biomarkers in this study are methane sulfonyl chloride (compound 6) and sulfurous acid hexyl octyl ether (compound 2) which are associated to be elevated in both cirrhotic and HCC patients, respectively.

#### The metabolization of ketonic compounds in liver

This was attributed to insulin resistance, which increased compound 5 and compound 10 in liver disorders. During lipolysis, unsaturated fats, triglycerides, and ketones were released [32]. As depicted in Fig 7, the ketonic molecule (also known as acetoin; compound 4) is elevated when the liver enzyme CYP2E1 is inhibited. In the current study, the *p*-values for these compounds are less than (*p*<0.05), showing a substantial increase in compounds 10 and 4 (fatty acids and ketones).

#### The metabolization of D-limonene (compound 9) in live

According to the results of this research, cirrhotic patients had a higher level of limonene in their breath than healthy individuals and HCC patients. Recently, Fernandez Del Rio et al. (2017 & 2020) found that restoring liver function with a liver transplant reduces compound 9 levels in the breath and restores them to normal [14, 33], although one study has stated that compound 9 level is elevated in the breath of cirrhotic patients due to CYP450 enzyme inactivity, as seen in Fig 7. In this study, the results support the above theory that variations in CYP450 enzyme activity between healthy and cirrhotic individuals increased the level of compound 9 in the exhaled breath of cirrhotic patients. Certain studies [34] examined VOCs in the breath of patients with liver disease, and certain VOCs have been proposed as biomarkers for cirrhosis. Sulfur compounds are associated with liver disease and are responsible for the distinct odor of patients’ breath known as Foetor Hepaticus. Limonene, methanol, and 2-pentanone have been identified as biomarkers of liver cirrhosis. These VOCs not only discriminated cases from controls but were also significantly different in a subset of transplant patients before and after liver transplantation. Limonene is converted in the liver by the P450 enzymes known as: CYP2C9 and CYP2C19 to the following metabolites: perillyl alcohol, trans-carveol, and trans-isopiperitenol. Cirrhosis patients have decreased levels of the enzyme CYP2C19, and these levels are adversely correlated with cirrhosis severity.

#### The metabolization of styrene (compound 7) in liver

Exogenous sources of styrene include plastics, cigarette smoke, exhaust fumes, and food [30]. As depicted in Fig 7, CYP2E1 is the primary enzyme responsible for styrene metabolism in humans. In this study, compound 7 levels are much greater in cirrhosis patients than in healthy individuals. In addition, the statistical results confirmed the idea that the amount of compound 7 is elevated in smokers with cirrhosis. According to the statistical data, the p-value of compound 7 in cirrhotic patients is less than 0.0001 (p<0.0001), confirming that CYP2E1 is dysfunctional in liver disease.

#### The metabolization of phenolic compounds in liver

Compound 1 is a prominent HCC breath biomarker associated with a phenolic family. The breakdown of tyrosine and tryptophan in the liver releases phenol and indole and subsequently binds to the albumin in the blood [35]. In liver disease, HCC, due to the loss of albumin, the indole and phenol lose their binding ability and cannot break down. So, the individuals with reduced liver function had larger quantities of free tyrosine and tryptophan. As a result, in the blood of these individuals, molecules like indole and phenol are raised, which shows that these biomarkers are the mediators of liver damage conditions. According to a recent study [36], MBMBP or MOCA compound oxidation is carried out by the liver enzyme P450. Several studies were carried out on the livers of rats in that study. Liver microsomes were important in the oxidation of MBMBP. In the instance of HCC, the inactivity of the cytochrome enzyme, p450, inhibited proper oxidation of MBMBP, resulting in a high concentration of MBMBP. The compound 4,4, Methylene bis (2,2 cholroaniline), known as MOCA, is the alternate name for the chemical MBMBP [37]. So, the elevated level of component 1 in this study, backs up the notion that albumin is reduced in the blood of HCC patients. The *p*-value of compound 1 identified in the statistical results of this investigation is <0.0001, indicating its high importance.

#### The metabolization of toluene (compound 4)

Compound 4 is metabolized in the liver by the P450 enzyme, which is essential for the biotransformation of many endogenous and foreign chemicals [38] whereas isoform P450, endogenous and exogenous substrates are all degraded by CYP2E1 enzyme. Compound 4 levels in the breath of HCC patients and healthy individuals differed significantly in this study. Higher levels of compound 4 in disease patients compared to healthy controls are indicative of abnormal activity of the liver enzyme CYP2E1.

#### Effect of confounding factors on the liver

In addition, confounding factors significantly impact the progression of liver cancer severity. Most of these confounding factors are smoking, age, gender, hepatitis B and C, and occasionally other environmental factors [39, 40]. In this study, we also examined the impact of confounding factors such as smoking, age, gender, and hepatitis B and C on the course of liver disease. Table 2 reveal the *p*-values of HCC and cirrhotic patients regarding smoking, hepatitis B, and hepatitis C are found to be less than 0.05 (p<0.05). The statistical study of drinking, age, and sex characteristics revealed a *p*-value greater than 0.05, indicating that these confounding factors are less significant.

## Supporting information

S1 File (DOCX)

S1 Data (XLSX)

## References

[1] Llovet J.M., et al.,Hepatocellular carcinoma. Nat Rev Dis Primers, 2021. 7(1):p.6 doi: 10.1038/s41572-020-00240-3 33479224 PMC13537433

[2] Di Bisceglie A.M., et al., Hepatocellular carcinoma. Annals of internal medicine, 1988. 108(3): p. 390–401. 2449110 10.7326/0003-4819-108-3-390

[3] McEvoy S.H., et al., Hepatocellular carcinoma: illustrated guide to systematic radiologic diagnosis and staging according to guidelines of the American Association for the Study of Liver Diseases. Radiographics, 2013. 33(6): p. 1653–1668. doi: 10.1148/rg.336125104 24108556

[4] Jee S.H., et al., Cigarette smoking, alcohol drinking, hepatitis B, and risk for hepatocellular carcinoma in Korea. Journal of the National Cancer Institute, 2004. 96(24): p. 1851–1856. doi: 10.1093/jnci/djh334 15601641

[5] Sherman M., et al., Screening for hepatocellular carcinoma: the rationale for the American Association for the Study of Liver Diseases recommendations. 2012, Wiley Online Library. p. 793–796. 10.1002/hep.25869 22689409

[6] Tarao K., et al., the Real Impact of liver cirrhosis on the development of hepatocellular carcinoma in various liver diseases—meta‐analytic assessment. Cancer medicine, 2019. 8(3): p. 1054–1065. 30791221 10.1002/cam4.1998 PMC6434205

[7] Leung T.-M. and Nieto N., CYP2E1 and oxidant stress in alcoholic and non-alcoholic fatty liver disease. Journal of hepatology, 2013. 58(2): p. 395–398. doi: 10.1016/j.jhep.2012.08.018 22940046

[8] Amal H., et al., The scent fingerprint of hepatocarcinoma: in-vitro metastasis prediction with volatile organic compounds (VOCs). International journal of nanomedicine, 2012. 7: p. 4135. doi: 10.2147/IJN.S32680 22888249 PMC3415321

[9] Kitiyakara T., et al., The detection of hepatocellular carcinoma (HCC) from patients’ breath using canine scent detection: a proof-of-concept study. Journal of breath research, 2017. 11(4): p. 046002. doi: 10.1088/1752-7163/aa7b8e 28649095

[10] Schmidt K. and Podmore I., Current challenges in volatile organic compounds analysis as potential biomarkers of cancer. Journal of biomarkers, 2015. 2015. doi: 10.1155/2015/981458 26317039 PMC4437398

[11] Arasaradnam R.P., et al., Breathomics—exhaled volatile organic compound analysis to detect hepatic encephalopathy: a pilot study. Journal of breath research, 2016. 10(1): p. 016012. doi: 10.1088/1752-7155/10/1/016012 26866470

[12] Pereira J., et al., Breath analysis as a potential and non-invasive frontier in disease diagnosis: an overview. Metabolites, 2015. 5(1): p. 3–55. doi: 10.3390/metabo5010003 25584743 PMC4381289

[13] Miekisch W., Schubert J.K., and Noeldge-Schomburg G.F., Diagnostic potential of breath analysis—focus on volatile organic compounds. Clinica Chimica Acta, 2004. 347(1–2): p. 25–39. doi: 10.1016/j.cccn.2004.04.023 15313139

[14] Fernández del Río R., Identification of volatile organic compounds in breath associated with liver disease and their potential applications for medical use. 2017, University of Birmingham.

[15] Boots A.W., et al., The versatile use of exhaled volatile organic compounds in human health and disease. Journal of breath research, 2012. 6(2): p. 027108. doi: 10.1088/1752-7155/6/2/027108 22621865

[16] Jia G.-S., et al., Using receiver operating characteristic curves to evaluate the diagnostic value of the combination of multislice spiral CT and alpha-fetoprotein levels for small hepatocellular carcinoma in cirrhotic patients. Hepatobiliary & Pancreatic Diseases International, 2017. 16(3): p. 303–309 doi: 10.1016/s1499-3872(17)60018-3 28603099

[17] Van Ruth S., et al., Prediction of the identity of fats and oils by their fatty acid, triacylglycerol and volatile compositions using PLS-DA. 2010. 118(4): p. 948–955.

[18] Rogers G., et al., Surveillance of cirrhosis for hepatocellular carcinoma: a systematic review and economic analysis. Health technology assessment (Winchester, England), 2007. 11(34): p. 1–206. 10.3310/hta11340 17767898

[19] Silva C., Passos M., and Camara J., Investigation of urinary volatile organic metabolites as potential cancer biomarkers by solid-phase microextraction in combination with gas chromatography-mass spectrometry. British journal of cancer, 2011. 105(12): p. 1894–1904. doi: 10.1038/bjc.2011.437 22085842 PMC3251876

[20] Stavropoulos G., van Munster K., Ferrandino G., Sauca M., Ponsioen C., van Schooten F. J., et al. (2021). Liver impairment—the potential application of volatile organic compounds in hepatology. Metabolites, 11(9), 618. doi: 10.3390/metabo11090618 34564434 PMC8471934

[21] Tanwar N. and Rahman K.F. Machine Learning in liver disease diagnosis: Current progress and future opportunities. in IOP Conference Series: Materials Science and Engineering. 2021. IOP Publishing.

[22] Wang L., et al., Volatile organic compounds as a potential screening tool for neoplasm of the digestive system: a meta-analysis. Scientific Reports, 2021. 11(1): p. 1–11. 34887450 10.1038/s41598-021-02906-8 PMC8660806

[23] Jalali M., et al., Oxidative stress biomarkers in exhaled breath of workers exposed to crystalline silica dust by SPME-GC-MS. Journal of research in health sciences, 2016. 16(3): p. 153. 27840344 PMC7191029

[24] Hershberger C. E., Rodarte A. I., Siddiqi S., Moro A., Acevedo‐Moreno L. A., Brown J. M., et al. (2021). Salivary metabolites are promising non‐invasive biomarkers of hepatocellular carcinoma and chronic liver disease. Liver cancer international, 2(2), 33–44. doi: 10.1002/lci2.25 34541549 PMC8447405

[25] Probert C.S., et al., Volatile organic compounds as diagnostic biomarkers in gastrointestinal and liver diseases. Journal of Gastrointestinal and Liver Disease, 2009. 18(3). 19795029

[26] Zhang Y., Guo L., Qiu Z., Lv Y., Chen G., & Li E. (2020). Early diagnosis of breast cancer from exhaled breath by gas chromatography‐mass spectrometry (GC/MS) analysis: A prospective cohort study. Journal of Clinical Laboratory Analysis, 34(12), e23526. doi: 10.1002/jcla.23526 33150682 PMC7755810

[27] Paradis V., et al., Identification of a new marker of hepatocellular carcinoma by serum protein profiling of patients with chronic liver diseases. Hepatology, 2005. 41(1): p. 40–47. doi: 10.1002/hep.20505 15690480

[28] Guo L., et al., Random-forest algorithm based biomarkers in predicting prognosis in the patients with hepatocellular carcinoma. Cancer cell international, 2020. 20(1): p. 1–12. 32565735 10.1186/s12935-020-01274-z PMC7302385

[29] Peled N., et al., An Update on the Use of Exhaled Breath Analysis for the Early Detection of Lung Cancer. Lung Cancer: Targets and Therapy, 2021. 12: p. 81. doi: 10.2147/LCTT.S320493 34429674 PMC8378913

[30] Bomhof M.R., et al., Histological improvement of non-alcoholic steatohepatitis with a prebiotic: a pilot clinical trial. European journal of nutrition, 2019. 58(4): p. 1735–1745. doi: 10.1007/s00394-018-1721-2 29779170

[31] Eggert T., et al., Fibrolamellar hepatocellular carcinoma in the USA, 2000–2010: A detailed report on frequency, treatment and outcome based on the Surveillance, Epidemiology, and End Results database. 2013. 1(5): p. 351–357. doi: 10.1177/2050640613501507 24917983 PMC4040774

[32] Farazi P.A. and DePinho R.A.J.N.R.C., Hepatocellular carcinoma pathogenesis: from genes to environment. 2006. 6(9): p. 674–687. 10.1038/nrc1934 16929323

[33] Ferrandino Giuseppe, Orf Isabel, Smith Rob, Calcagno Marzia, Anita Kaur Thind, Irene Debiram-Beecham, et al. "Breath biopsy assessment of liver disease using an exogenous volatile organic compound—toward improved detection of liver impairment." Clinical and translational gastroenterology 11, no. 9 (2020). doi: 10.14309/ctg.0000000000000239 33094960 PMC7498135

[34] Gopas J., et al., Urine volatile organic compounds composition in mice bearing breast and melanoma tumors: effect of a low-protein diet. 2018. 8(1): p. 1–13

[35] O’Hara ME, Fernández Del Río R, Holt A, Pemberton P, Shah T, Whitehouse T, et al. Limonene in exhaled breath is elevated in hepatic encephalopathy. J Breath Res. 2016 Nov 21;10(4):046010. doi: 10.1088/1752-7155/10/4/046010 ; PMCID: PMC5500822. 27869108 PMC5500822

[36] Pathiratne A., In vitro metabolism of benzene, toluene, and xylene in rat liver. 1985, North Dakota State Univ., Fargo (USA).

[37] Szaefer H., et al., Effect of naturally occurring plant phenolics on the induction of drug metabolizing enzymes by o-toluidine. Toxicology, 2003. 186(1): p. 67–77 doi: 10.1016/s0300-483x(02)00615-7 12604171

[38] Butler MA, Guengerich FP, Kadlubar FF. Metabolic oxidation of the carcinogens 4-aminobiphenyl and 4,4’-methylene-bis(2-chloroaniline) by human hepatic microsomes and by purified rat hepatic cytochrome P-450 monooxygenases. Cancer Res. 1989 Jan 1;49(1):25–31. . 2908851

[39] Sabbioni G., & Neumann H. G. (1990). Quantification of haemoglobin binding of 4, 4′-methylenebis (2-chloroaniline) (MOCA) in rats. Archives of toxicology, 64, 451–458. doi: 10.1007/BF01977626 1703404

[40] Chapman D.E., et al., Metabolism and covalent binding of [14C] toluene by human and rat liver microsomal fractions and liver slices. 1990. 18(6): p. 929–936. 1981539

