# Supplementary material for: Novel breath biomarkers identification for early detection of hepatocellular carcinoma and cirrhosis using ML tools and GCMS
Source: PLoS One. 2023 Nov 15;18(11):e0287465. doi: 10.1371/journal.pone.0287465 (PMC10651033; doi:10.1371/journal.pone.0287465)
Supplement: S1 File — (DOCX) [file pone.0287465.s001.docx]

**Supplementary Information**

**Novel breath biomarkers identification for early detection of hepatocellular carcinoma and cirrhosis using ML tools and GCMS.**

Noor ul Ain Nazir^1, 2^, Muhammad Haroon Shaukat^3^, Ray Luo^4,5^, and Shah Rukh Abbas^1*^

^1^Atta-Ur-Rahman School of Applied Biosciences, National University of Sciences and Technology (NUST), Islamabad, 44000, Pakistan. ^2^Department of Electrical Engineering and Computer Science, The Henry Samueli School of Engineering, University of California, Irvine, CA, 92617, United States. ^3^National Agriculture and Research Center (NARC), Islamabad, 44000, Pakistan. ^4^Departments of Chemical and Biomolecular Engineering, Materials Science and Engineering, and Biomedical Engineering, the University of California, Irvine, CA, 92617, ^5^Department of Molecular Biology and Biochemistry, School of Biological Sciences, University of California, Irvine, CA, 92617, United States.

***Corresponding Author**

**Dr. Shah Rukh Abbas, Ph.D.** Atta-Ur-Rahman School of Applied Biosciences,

National University of Sciences and Technology, Islamabad, 44000, Pakistan.

**Email:** [sabbas@asab.nust.edu.pk](mailto:sabbas@asab.nust.edu.pk)

**Phone number:** +92-335 5449622

**Author Contributions:** N.U.A.N., and S.R.A., had full access to all the data in the study and take responsibility for the integrity of the data and the accuracy of the data analysis. ***Concept and Design:*** S.R.A and N.U.A.N. are responsible for the concept and design of this project. ***Acquisition, analysis, or interpretation of data:*** N.U.A.N., & M.H.S., have contributed to the analysis & interpretation of data. ***Drafting of the manuscript:*** N.U.A.N., wrote the final draft which was proofread by R.L., and S.R.A. ***Statistical analysis:*** N.U.A.N., and M.H.S

**Legends**

S1 Fig. PCA analysis. Scree plots (A) HCC vs control (B) Cirrhosis & control (C) HCC vs cirrhosis

S2 Fig. ROC curves for the diagnostic accuracy detection**.** Significant HCC and cirrhotic breath biomarkers were screened out through ML models and GCMS analysis.

S1 Table. Concentration and *p* values of HCC & Cirrhotic breath biomarkers (VOCs) through GCMS analysis.

S1 Text. Sampling questionnaire

S2 Text. IRB Performa

**S1 Fig**


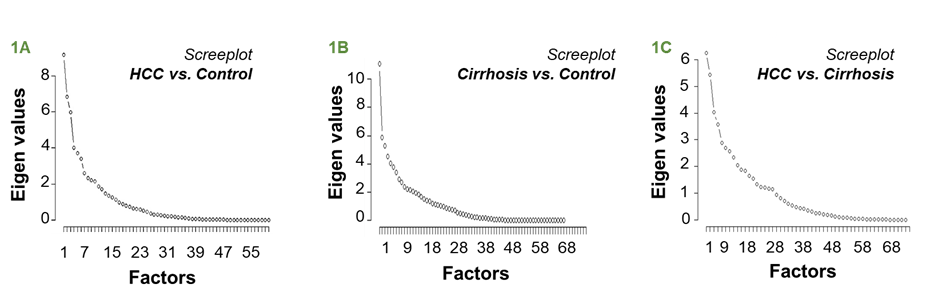


**S2 Fig**


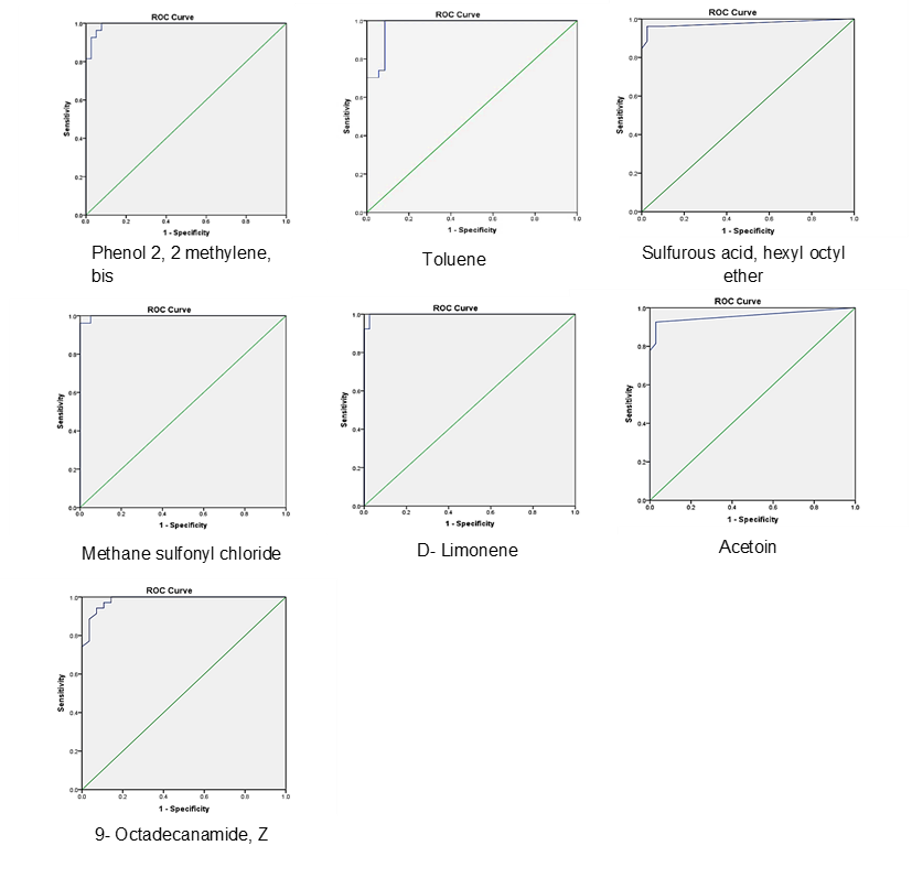


S1 Table

| **Sr No** | **Metabolites** | **Disease** | **RT (mints)** | **P <values** | **Identification methods** | **Ppm values** |
| --- | --- | --- | --- | --- | --- | --- |
| 1 | D limonene | Cirrhosis | 8 | <0.001 | GCMS | 800ppm |
| 2 | Methane sulfonyl chloride | Cirrhosis | 7.69 | <0.001 | GCMS | 1200ppm |
| 3 | Acetoin | Cirrhosis | 6.95 | <0.005 | GCMS | 700ppm |
| 4 | Styrene | Cirrhosis |  | <0.05 | GCMS | 1000ppm |
| 5 | Di n octyl phthalate | Cirrhosis | 32.37 | <0.05 | GCMS | 100ppm |
| 6 | Toluene | HCC | 4.87 | <0.05 | GCMS | 500ppm |
| 7 | Phenol 2, 2 methylene, Bis | HCC | 27.57 | <0.001 | GCMS | 2100ppm |

**S1 Text**

**Sampling Questionnaire**

Thank you very much for your time and participation.

1. Name:
2. Age : <30 30-40 40-60 >60
3. Gender: Male Female

1. Region/Area: Punjab Sindh Baluchistan KPK Islamabad

1. Education: Elementary School High School College Post graduate
2. Marital Status: Single Married Divorced/ Separated
3. How often do you have a drink containing alcohol?
4. Never
5. Monthly or less
6. 2-4 times a month
7. 2-3 times a week
8. 4 or more times a week

1. How many standards drinks containing alcohol do you have on typical day?
2. 1 or 2
3. 3 or 4
4. 5 or 6
5. 7 to 9
6. 10 or more
7. Do you smoke?

A). Yes b). No

1. How often do you smoke in a day? (If Yes in question 9)
2. Less than 5
3. 5 to 10
4. 11 to 20
5. 21 to 30
6. 31 or more
7. History of testing for Hepatitis B?

A).Yes and was positive b). Yes and was negative c). No d). I don’t know

1. History of testing for Hepatitis C?

A).Yes and was positive b). Yes and was negative c). No d). I don’t know

1. Have you ever vaccinated against Hepatitis B?

A). Yes, I have received and completed. B). Yes I have started and may have finished c). No.

1. Have you ever vaccinated against Hepatitis C?

A). Yes, I have received and completed. B). Yes I have started and may have finished c). No.

1. Family member with Hepatitis B?

A). Yes b). No c). I don’t know

1. Family member with Hepatitis C?

A). Yes b). No c). I don’t know

1. Do you exercise daily?

A). Yes b). No

18. How often do you exercise? (If yes in question 17)

A). Daily b). Three times in a week c). Twice in a week d). Once in month.

19. Have you been diagnosed with Liver cirrhosis?

A). Yes b). No

20. Have you been diagnosed with hepatocellular carcinoma (HCC)?

A). Yes b). No

21. For how many years did you diagnose with HCC?

A). Less than 1 year b). 1 to 2 years c). 3 to 4 years d). I don’t know

22. On which stage of HCC, did you diagnose?

A). 1^st^ b). 2^nd^ c) 3^rd^ d) 4th e) I don’t know

23. Do you receive any chronic medication?

A). Yes b). No

24. Did you have operations before?

A). Yes b). No.

25. Are you satisfied with current diagnostic/screening techniques?

A). Yes b). No c) I don’t know

26. Are you patient of any disease other than HCC and liver cirrhosis?

A). Yes (Name :---------------------------)

B). No

**S2 Text**

**IRB Performa**

(To be completed by the PI)

1. Project title: **Early detection of Hepatocellular carcinoma (HCC) through breath biosensor**
2. Name of the Principal Investigator: Dr. Shah Rukh Abbas
3. Name of the Co-PI (if any): N/A
4. Name of the Department: Atta ur Rehman School of Applied Biosciences (ASAB)
5. Designation: Assistant Professor

| S# | Description | Comments |
| --- | --- | --- |
| 1 | Qualification, Expertise and Scientific  Caliber of the Principal Investigators | PI: PhD in Biotechnology  Expertise: Nanotechnology, biosensors, nanoconstructs for drug delivery and imaging applications. (Diagnostics and therapeutics).  Co-PI:  Expertise: |
| 2 | Proposed Goals of the Study | 1. To construct an electrochemical biosensor for screening of Hepatocellular carcinoma (HCC). 2. To identify and quantify Volatile Organic Compound (VOCs) biomarkers in the breath and serum samples of HCC through GC-MS. |
| 3 | Subject Selection | The HCC patients, Liver cirrhosis and healthy individuals will be selected for breath and serum samples. |
| 4 | Selection Criteria of Subjects | Male and females both will be selected. The mean age for the selection will be 35-50. Fasting and non-fasting, Smokers and non-smokers both will be be selected. Each subject will fill up a questioner and content form prior to sampling. |
| 5 | Potential Problems | There is no potential problem |
| 6 | Research Design and Methods | **Methodology for Breathe and Serum samples collection and profiling of these markers:**  1. The breath samples will be collected in teddlar bags. About 106 breath samples and 106 serum samples from both healthy individuals and HCC patients will be selected.  About 243 patients of liver cirrhosis and 100 healthy individuals will be selected  2. About 4 ml of blood samples of fasting, no fasting, smokers and nonsmokers will be collected in 5mL gel based BD tubes. Serum will separated by centrifugation at 4000 rpm for 10 mints. Stored these samples in freezer at -80ͦ C.  3. The breath samples and serum samples will further process through GC-MS for the quantification of biomarkers in the samples. |
| 7 | Potential Benefits of the Study | It will be first time in the Pakistan to screen the HCC at early stages through Breath samples.  Moreover, the screening of HCC through biosensor will be noninvasive, less time consuming and will give high accuracy as compared to current techniques.  This screening method will be economical. As current techniques are much expensive. So it will be easy and economical for patients. |
| 8 | Risks of the Study | There is no potential risk of study. All procedures including sampling will be handled properly according to the standard experimental guidelines. |
| 9 | Management of Risks | There is no potential risk; All procedures including sampling will be performed wearing Personal Protective Equipment throughout the study. Sampling will be done by nurses and in the presence of expert medical professionals at Shifa and PIMS (Our collaborators). |
| 10 | Assessment of Risk | There are no serious threats associated to proposed study. The experiments in general, will be monitored regularly to avoid any accidental risk. |
| 11 | Confidentiality | I own this project and I will acknowledge all who provide technical and/or material support. |
| 12 | Conflict of Interest | No conflict of interest |

1. Impact of proposed research on training of manpower and institutional capacity building or on local industry:

The proposed study will look in to the VOC biomarker identification in breath and serum samples of HCC patients. It also will further use this information in fabricating a biosensor to screen the population for HCC, non-invasively. The project will help creating first breath and serum VOC profiling of HCC patients to identify markers to be used. Furthermore the biosensor is aiming to screen HCC at an early stage in less time making it more advantageous over existing techniques. This technique, once translated would be helpful in managing HCC mortality and morbidity ratios by screening HCC at an early stage, and fast without the need of any technical personnel.

Signature: ………………………

Name:…………………………....

Designation:…………………….

Address: ………………………...

Phone: …………………………..

Email:…………………………… Dated ……………………………
